# Characteristics associated with successful foodborne outbreak investigations involving United States retail food establishments (2014–2016)

**DOI:** 10.1017/S0950268823000237

**Published:** 2023-03-20

**Authors:** Meghan M. Holst, Adam Kramer, Edward Rickamer Hoover, Daniel Dewey-Mattia, James Mack, Tracy Hawkins, Laura G. Brown

**Affiliations:** 1 Department of Environmental Health Science and Practice, Centers for Disease Control and Prevention, National Center for Environmental Health, 4770 Buford Highway, Atlanta, Georgia 30341, USA; 2 Department of Foodborne, Waterborne, and Environmental Diseases, Centers for Disease Control and Prevention, National Center for Emerging and Zoonotic Infectious Diseases, 1600 Clifton Road, Atlanta, GA 30329, USA; 3 Wisconsin Department of Health Services, 1 West Wilson Street, Madison, Wisconsin 53702, USA; 4 Indiana Department of Health, Food Protection Division, 2 North Meridian Street, Indianapolis, Indiana 46204, USA

**Keywords:** Environmental management, epidemiology, food-borne infections, food safety, outbreaks

## Abstract

This study examined relationships between foodborne outbreak investigation characteristics, such as the epidemiological methods used, and the success of the investigation, as determined by whether the investigation identified an outbreak agent (i.e. pathogen), food item and contributing factor. This study used data from the Centers for Disease Control and Prevention's (CDC) National Outbreak Reporting System and National Environmental Assessment Reporting System to identify outbreak investigation characteristics associated with outbreak investigation success. We identified investigation characteristics that increase the probability of successful outbreak investigations: a rigorous epidemiology investigation method; a thorough environmental assessment, as measured by number of visits to complete the assessment; and the collection of clinical samples. This research highlights the importance of a comprehensive outbreak investigation, which includes epidemiology, environmental health and laboratory personnel working together to solve the outbreak.

## Introduction

Foodborne illnesses are a significant concern in the U.S. The Centers for Disease Control and Prevention (CDC) estimates that 48 million people get sick, 128 000 are hospitalised, and 3000 die from foodborne illness annually in the U.S [1]. The estimated annual health-related costs of foodborne illnesses are around $90 million [2]. Many foodborne illnesses are sporadic; however, some are associated with outbreaks; an average of 823 foodborne illness outbreaks occurs in the U.S. every year [3].

State and local health departments are typically responsible for investigating foodborne outbreaks at retail food establishments in their jurisdictions. During their investigations, they focus on collecting three types of data: epidemiologic information about illness cases and route of exposure (i.e. food item contaminated with the foodborne pathogen), laboratory information about the foodborne pathogen (i.e. agent) and environmental health information about how the agent contaminated the food (i.e. contributing factors). A contributing factor is defined as a food preparation practice that leads to a food getting contaminated with pathogens or that lead to pathogens growing in food. There are 30 contributing factors that fall into 3 categories: contamination, proliferation and survival [4]. For example, a 2016 investigation of an outbreak in which people became ill with foodborne illness symptoms after eating at a restaurant identified the agent as *Campylobacter jejuni*, the food item as chicken liver mousse, and the contributing factor as pathogen survival resulting from insufficient time or temperature control during cooking of the mousse (i.e. undercooking) [5].

These investigation data are valuable to public health in several ways. First, they can be used to control and end the immediate outbreak being investigated. For example, in the chicken liver mousse outbreak described above, once the agent, food item and contributing factor were identified, health department staff worked with restaurant staff to improve their chicken liver mousse cooking practices to end the immediate outbreak. Second, analysis of these data at the aggregate level can inform efforts to prevent future outbreaks. For example, data from investigations of multiple chicken liver outbreaks informed the development of national guidance and educational materials for restaurants on preventing *Campylobacter* illnesses associated with chicken liver; these materials focused on proper cooking of chicken livers [6]. Third, because sporadic foodborne illnesses can have the same epidemiology as foodborne outbreaks, these data can inform efforts to prevent sporadic illnesses. For example, in an effort to reduce sporadic *Campylobacter* illnesses, the audience for the previously mentioned guidance on proper cooking of chicken livers included not only restaurants, but also consumers [5].

Despite the public health value of these outbreak investigation data, advances in laboratory technology that identifies foodborne pathogens, and CDC efforts to support health departments in their investigational efforts [7], investigators do not always collect these data. The most recent CDC data indicate that 47% of reported foodborne outbreaks had an identified agent, 43% had an identified food item, and 26% had an identified contributing factor [8, 9]. There are multiple factors that influence investigators’ success in identifying these facets of outbreaks, including available resources, investigator training and skill level, cooperation from outbreak establishment staff, and ill persons’ ability to remember what they ate [10]. Investigation characteristics, such as epidemiological methods used and timeliness of initiation, may also impact investigation outcomes. Indeed, recent research indicates that some characteristics of outbreak investigations are associated with successful identification of outbreak contributing factors [11].

We conducted the present study to further explore the relationship between outbreak investigation characteristics and investigation outcomes at retail food establishments. Specifically, we examined associations between outbreak investigation characteristics and the success of the investigation, as determined by how many of the three facets (agent, food item and contributing factor) the investigation identified. We present percentages of achieving different outbreak investigation success levels (number of facets identified) depending on the investigation's characteristics.

## Materials and methods

We obtained data from two CDC foodborne outbreak reporting systems: the National Outbreak Reporting System (NORS) and the National Environmental Assessment Reporting System (NEARS) [12]. Health departments report data from their foodborne outbreak investigations to these systems [13]. The data obtained included relevant outbreak, establishment and investigation characteristics. An outbreak is defined as two or more cases of a similar illness associated with a common exposure, such as a shared food, venue or experience [13].

### National outbreak reporting system (NORS)

All state health departments report epidemiologic and laboratory data from their foodborne outbreak investigations to NORS [14]. Typically, epidemiology or communicable disease control programs within health departments collect and report these data. We obtained NORS data on whether an outbreak agent was identified, method of epidemiological investigation (interviews with ill people, case–control study, cohort study), the type of samples taken (clinical [i.e. stool samples], environmental [i.e. food, surfaces]), per cent of primary cases seeking healthcare and date of first illness onset. For the epidemiological investigation method variable, investigators reported all methods used in the investigation. We created a new method variable that identified the most rigorous investigation method for each outbreak, with cohort study considered the most rigorous, followed by case–control study and interviews of ill people [15].

### National environmental assessment reporting system (NEARS)

Some state and local health departments choose to report data from the environmental health component or environmental assessment, of their foodborne outbreak investigations to NEARS. Typically, environmental health programs within health departments collect and report these data. This environmental assessment is designed to describe the environment in which the outbreak occurred and identify the factors and antecedents contributing to outbreaks. For health departments that report data to NEARS, this assessment usually includes a standardised interview with the outbreak establishment manager, and an environmental observation of the establishment, including records, menus and food preparation practices (conducted in no particular order). NEARS data on the outbreak establishments obtained for this analysis include whether an outbreak food item was identified, number of meals served daily, establishment type (restaurant *vs.* other), menu type (American *vs.* International) and food preparation complexity (complex [at least one food item requires a process, like cooking, that eliminates pathogens and additional processes like cooling and reheating] *vs.* other). Number of meals served daily, establishment type and menu type were dichotomised for the analysis. Menu type was dichotomised into two categories: American and International, which included Chinese, Thai, Japanese, French, Italian and Mexican cuisines. NEARS data on the outbreak investigation included: date the establishment was identified as a suspected outbreak location, date of first contact with the establishment, number of visits made to the outbreak establishment to complete the environmental assessment, whether an interview was conducted with the outbreak establishment manager, whether an outbreak establishment observation was conducted, and whether an outbreak contributing factor was identified. These data were determined by the NEARS investigator based on their environmental assessment activities, with the exception of the number of meals served daily; this variable was collected through an interview with the outbreak establishment manager.

### Outbreak

From January 1, 2014 through December 31, 2016, 16 state and local health departments reported 404 foodborne illness outbreaks to NEARS. Of the 404 NEARS foodborne illness outbreaks, we matched 334 (82.7%) with a NORS outbreak record; matches were based on outbreak identification numbers reported to each system and manual review of pertinent outbreak characteristics. Of the 334 matched outbreaks, we excluded 9 (2.7%) as they had exposures that took place at multiple locations. Finally, we excluded outbreaks reported by three health departments with an insufficient number of reported outbreaks (<8) to meet the needs of our analyses. The final data set, consisting of 306 outbreaks, included data from Connecticut, Minnesota, New York City, New York State (excluding New York City), Rhode Island, Tennessee, Washington and Wisconsin.

### Analysis

We first defined and created our variable of interest, outbreak investigation success. This variable included three levels of investigation success: completely successful, partially successful and unsuccessful. The degree of success is based upon identification of three components: an agent (suspected or confirmed), a food item (suspected or confirmed) and a contributing factor. A completely successful foodborne outbreak investigation identified all three components, a partially successful investigation identified one or two components, and an unsuccessful investigation did not identify any of the components. We also created several investigation variables based on existing data; these included: number of days from establishment identification to first contact with the establishment, number of days from establishment identification to manager interview, number of days from establishment identification to establishment observation, and number of days from first illness onset to establishment identification.

We calculated overall descriptive statistics for outbreak and outbreak investigation characteristics by investigation success (Table 1). Individual multiple ordinal logistic regression served as our principal analysis to estimate the probability of investigation success. Several model diagnostics were conducted for each explanatory characteristic including the Hosmer–Lemeshow test of goodness-of-fit and score test of proportional odds. We also looked for decreases in Akaike information criterion (AIC) in comparison to the baseline model (which included only data collection site); a decrease indicates a better model fit. Multiple ordinal logistic regression identified a common set of characteristics associated with a completely successful outbreak investigation (completely successful *vs.* partially or unsuccessful) and at least a partially successful investigation (completely or partially successful *vs.* unsuccessful). The outbreak establishment characteristics investigated in this analysis were: number of meals served daily, establishment type, menu type, food preparation process and per cent of cases who sought healthcare. The outbreak investigation characteristics included in this analysis were: epidemiology investigation method used, sample type, number of visits to complete the environmental assessment, time from identification of establishment to first contact, time from identification of establishment to manager interview, time from identification of establishment to observation and time from first illness onset to identification of establishment. Explanatory variables were chosen because they are known to be associated with food safety at retail food establishments [11, 16–18].

**Table 1.** Outbreak establishment and investigation characteristics by investigation success level – National Environmental Assessment Reporting System, 2014–2016

		Outbreaks with		
	All outbreaks (*n* = 306, 100%)	Completely successful investigations^a^ (*n* = 106, 34.6%)	Partially successful investigations^a^ (*n* = 177, 57.8%)	Unsuccessful investigations^a^ (*n* = 23, 7.5%)
Establishment characteristics				
Number of meals served daily (*n* = 245)^b^				
⩽200	129 (52.6)	40 (51.9)	76 (51.7)	13 (61.9)
>200	116 (47.4)	37 (48.1)	71 (48.3)	8 (38.1)
Establishment type (*n* = 306)				
Restaurant	262 (85.6)	86 (81.1)	156 (88.1)	20 (87.0)
Other	44 (14.4)	20 (18.9)	21 (11.9)	3 (13.0)
Menu type (*n* = 306)				
American	172 (56.2)	63 (59.4)	101 (57.1)	8 (34.8)
International	134 (43.8)	43 (40.6)	76 (42.9)	15 (65.2)
Food preparation process (*n* = 306)				
Complex	279 (91.2)	99 (93.4)	160 (90.4)	20 (87.0)
Other	27 (8.8)	7 (6.6)	17 (9.6)	3 (13.0)
Per cent of primary cases who sought healthcare (*n* = 279)^b^^,^^c^				
0	121 (43.4)	37 (37.0)	73 (45.3)	11 (61.1)
1–20	107 (38.3)	40 (40.0)	61 (37.9)	6 (33.3)
>20	51 (18.3)	23 (23.0)	27 (16.8)	1 (5.6)
Outbreak investigation characteristics				
Epidemiology investigation method (*n* = 306)^c^				
None	24 (7.8)	5 (4.7)	13 (7.3)	6 (26.1)
Interviews with ill people	114 (37.3)	50 (47.2)	57 (32.2)	7 (30.4)
Case–control study	114 (37.3)	34 (32.1)	77 (43.5)	3 (13.0)
Cohort study	54 (17.6)	17 (16.0)	30 (17.0)	7 (30.5)
Sample type (*n* = 306)^c^				
None	157 (51.3)	44 (41.5)	95 (53.6)	18 (78.3)
Environmental	18 (5.9)	6 (5.7)	9 (5.1)	3 (13.1)
Clinical	98 (32.0)	39 (36.8)	58 (32.8)	1 (4.3)
Both	33 (10.8)	17 (16.0)	15 (8.5)	1 (4.3)
Number of visits to complete the environmental assessment (*n* = 306)				
0	80 (26.1)	16 (15.1)	51 (28.8)	13 (56.5)
1	174 (56.9)	64 (60.4)	103 (58.2)	7 (30.4)
2+	52 (17.0)	26 (24.5)	23 (13.0)	3 (13.1)
Time from identification of establishment to first contact (*n* = 306)				
Same day	208 (68.0)	67 (63.2)	126 (71.2)	15 (65.2)
1–2 days	74 (24.2)	28 (26.4)	39 (22.0)	7 (30.4)
3+ days	24 (7.8)	11 (10.4)	12 (6.8)	1 (4.4)
Time from identification of establishment to manager interview (*n* = 262)^b^				
Same day	59 (22.5)	11 (14.1)	42 (25.8)	6 (28.6)
1–2 days	52 (19.9)	15 (19.2)	30 (18.4)	7 (33.3)
3+ days	151 (57.6)	52 (66.7)	91 (55.8)	8 (38.1)
Time from identification of establishment to establishment observation (*n* = 291)^b^				
Same day	148 (50.8)	48 (48.5)	89 (51.7)	11 (55.0)
1–2 days	75 (25.8)	28 (28.3)	41 (23.9)	6 (30.0)
3+ days	68 (23.4)	23 (23.2)	42 (24.4)	3 (15.0)
Time from 1st illness onset to identification of establishment (*n* = 284)^b^^,^^c^				
Same day	11 (3.9)	4 (4.0)	5 (3.0)	2 (11.1)
1–2 days	81 (28.5)	25 (24.7)	50 (30.3)	6 (33.3)
3+ days	192 (67.6)	72 (71.3)	110 (66.7)	10 (55.6)

a A completely successful outbreak investigation identified an agent, a food item and a contributing factor. A partially successful outbreak investigation identified 1 or 2 of either an agent, a food item or a contributing factor. An unsuccessful outbreak investigation did not identify an agent, a food item or a contributing factor.

b Values may not add up to the total number of observations if the manager interview or establishment observation were not conducted. If the interview and observation were conducted, it is possible that the manager was unable to answer the question or that the investigator could not make the observation.

c Variable collected by the National Outbreak Reporting System.

We controlled for the eight health departments reporting the outbreaks to account for differences across jurisdictions, such as differences in regulations, investigation practices and regional food preferences. We present results in terms of the percentages of a completely successful (Table 2) and at least a partially successful (Table 3) foodborne outbreak investigation for characteristics with a statistical significance of *P* ≤ 0.05. Outbreak frequencies at each data collection site are presented in supplementary table 1. Significance for each characteristic was determined by a type 3 likelihood ratio test. For characteristics significantly associated with investigation success, we conducted post-hoc pairwise comparisons between the difference of estimated percentages. We calculated adjusted *P*-values using a Scheffé test. Maximum likelihood estimates, least square means and odds ratios for each significant explanatory variable are presented in supplementary tables 2–10. All analyses were conducted using SAS 9.4.

**Table 2.** Estimated percentages of conducting a completely successful foodborne outbreak investigation^a^ by investigation characteristic (*N* = 306)

Investigation characteristic	%^b^	Difference of percentage points	adj. *P*-value^c^
Epidemiology investigation method (main effect *P* = 0.05)			
Cohort study vs.	37.7	–	–
Interviews with ill people	33.9	3.8	0.97
Case–control study	27.3	10.4	0.69
No method	11.4	26.3	0.05
Interviews with ill people vs.	33.9	–	–
Case–control study	27.3	6.6	0.80
No method	11.4	22.5	0.04
Case–control study vs.	27.3	–	–
No method	11.4	15.9	0.22
Number of environmental assessment visits (main effect *P* = 0.02)			
2+ visits vs.	43.7	–	–
1 visit	31.3	12.4	0.30
No visit	18.1	25.6	0.01
1 visit vs.	31.3	–	–
No visit	18.1	13.2	0.10
Sample type (main effect *P* < 0.01)			
Clinical and environmental vs.	43.9	–	–
Only clinical	39.4	4.5	0.97
Only environmental	31.3	12.6	0.84
None	21.2	22.7	0.07
Only clinical vs.	39.4	–	–
Only environmental	31.3	8.1	0.93
None	21.2	18.2	<0.01
Only environmental vs.	31.3	–	–
None	21.2	10.1	0.79

a A completely successful outbreak investigation identified an agent, a food item and a contributing factor.

b Estimated percentages derived from least square means found in Supplementary Tables 3,6, and 9.

c *P*-value adjusted for multiple comparisons using a Scheffé test.

**Table 3.** Estimated percentages of conducting at least a partially successful foodborne outbreak investigation^a^ by investigation characteristic (*N* = 306)

Investigation characteristic	%^b^	Difference of percentage points	adj. *P*-value^c^
Epidemiology investigation method (main effect *P* = 0.05)			
Cohort study vs.	95.7	–	–
Interviews with ill people	94.9	0.8	0.97
Case–control study	93.2	2.5	0.69
No method	82.5	13.2	0.05
Interviews with ill people vs.	94.9	–	–
Case–control study	93.2	1.7	0.80
No method	82.5	12.4	0.04
Case–control study vs.	93.2	–	–
No method	82.5	10.7	0.22
Number of environmental assessment visits (main effect *P* = 0.02)			
2+ visits vs.	96.6	–	–
1 visit	94.3	2.3	0.30
No visit	89.0	14.1	0.01
1 visit vs.	94.3	–	–
No visit	89.0	5.3	0.10
Sample type (main effect *P* < 0.01)			
Clinical and environmental vs.	96.8	–	–
Only clinical	96.1	0.7	0.97
Only environmental	94.6	2.2	0.84
None	91.1	5.7	0.07
Only clinical vs.	96.1	–	–
Only environmental	94.6	1.5	0.93
None	91.1	5.0	<0.01
Only environmental vs.	94.6	–	–
None	91.1	3.5	0.79

a A foodborne outbreak investigation is at least partially successful when one of more facets (i.e. agent, food or contributing factor) has been identified.

b Estimated percentages derived from least square means found in Supplementary Tables 3,6, and 9.

c *P*-value adjusted for multiple comparisons using a Scheffé test.

## Results

### Outbreak establishment and investigation characteristics

Most reported outbreaks occurred at a restaurant (85.6%). The most common epidemiology investigation methods used were interviews of ill people (37.3%) and case–control studies (37.3%), followed by cohort studies (17.6%); lack of an epidemiology investigation was rare (7.8%). Samples were collected in about half of the investigations (48.7%); clinical samples (32.0%) and both clinical and environmental samples (10.8%) were more frequently collected than only environmental samples (5.9%). Investigators most often made one visit to the outbreak establishment to complete the environmental assessment (56.9%) (Table 1).

Investigators most often made first contact with the outbreak establishment after it was identified as a suspected outbreak location on the same day of identification (68.0%). They less often made contact 1–2 days (24.2%) or 3 or more days (7.8%) after identification. Investigators most frequently interviewed the establishment manager 3 or more days (57.6%) after establishment identification, followed by the same day (22.5%) and 1–2 days after (19.9%). The establishment observation frequently occurred the same day (50.8%) the establishment was identified and less frequently 1–2 days (25.8%) and 3 or more days (23.4%) after identification of the establishment. Typically, 3 or more days (67.6%) elapsed between the first illness onset and establishment observation, followed by 1–2 days (28.5%) and the same day (3.9%) (Table 1). Descriptive data on additional characteristics are listed in Table 1.

### Outbreak investigation success

Most outbreaks (82.0%, 251) had an identified agent; 52.6% (161) had an identified food item, and 82.0% (251) had an identified contributing factor. A third of outbreaks (34.6%, 106) had all three of these facets identified. Over half of outbreaks (57.8%, 177) had one or two of these facets identified; 7.5% (23) had none identified.

### Relationships between outbreak characteristics and investigation success

Individual multiple ordinal regressions, controlling for data collection site, identified three characteristics that were significantly associated with an outbreak investigation success (Tables 2 and 3). These characteristics were epidemiology investigation method (*P* = 0.05), number of visits to complete the environmental assessment (*P* = 0.02) and types of samples taken during the investigation (*P* < 0.01).

### Epidemiology investigation method

Pairwise comparisons showed that the probability of a completely successful investigation was significantly higher (*P* = 0.05) when a cohort study was conducted (37.7%) and when interviews with ill people were conducted (33.9%) than when no epidemiological method was conducted (11.4%) (Table 2). Pairwise comparisons showed the same pattern for the probability of an at least partially successful investigation; the probability was significantly higher (*P* = 0.05) when a cohort study was conducted (95.7%) and when interviews with ill people were conducted (94.9%) than when no epidemiological method was conducted (82.5%) (Table 3). Maximum likelihood estimates, least square means and odds ratios are available in supplementary tables (Supplementary Tables 2–4).

### Number of visits to complete the environmental assessment

Pairwise comparisons showed that the probability of a completely successful investigation was significantly higher (*P* = 0.02) when 2 or more visits were made to complete the environmental assessment (43.7%) than when no visits were made (18.1%) (Table 2). Pairwise comparisons showed the same pattern for the probability of an at least partially successful investigation; the probability was significantly higher (*P* = 0.02) when 2 or more visits were made to complete the environmental assessment (96.6%) than when no visits were made (89.0%) (Table 3). Maximum likelihood estimates, least square means and odds ratios are available in supplementary tables (Supplementary Tables 5–7).

### Sample type

Pairwise comparisons showed that the probability of a completely successful investigation was significantly higher (*P* = 0.01) when only clinical samples were taken (39.4%) than when no samples were taken (21.2%) (Table 2). Pairwise comparisons showed the same pattern for the probability of an at least partially successful investigation; the probability was significantly higher (*P* = 0.01) when only clinical samples were taken (96.1%) than when no samples were taken (91.1%) (Table 3). Maximum likelihood estimates, least square means and odds ratios are available in supplementary tables (Supplementary Tables 8–10).

The model diagnostics were investigated for each significant characteristic including a decrease in AIC for model fit in comparison to the baseline model containing only data collection site, Hosmer–Lemeshow (HL) test of goodness-of-fit and score test of proportional odds. The inclusion of epidemiology investigation method decreased AIC by 1.8236, HL between completely successful and less than completely successful investigation was not statistically significant (*P* = 0.944), as well as between at least partially successful and unsuccessful investigation (*P* = 0.970), and the score test of proportional odds was not statistically significant (*P* = 0.356). The inclusion of the number of environmental assessments decreased AIC by 2.469, HL between completely successful and less than completely successful investigation was not statistically significant (*P* = 0.683), as well as between at least partially successful and unsuccessful investigation (*P* = 0.612), and the score test of proportional odds was not statistically significant (*P* = 0.727). The inclusion of sample type decreased AIC by 5.0886, HL between completely successful and less than completely successful investigation was not statistically significant (*P* = 0.944), as well as between at least partially successful and unsuccessful investigation (*P* = 0.970), and the score test of proportional odds was not statistically significant (*P* = 0.370).

## Discussion

Identifying an agent, food item and contributing factor can help investigators educate retail food establishment management and workers on gaps in their food safety practices and guide future outbreak investigations. For the outbreaks in this study, investigators were most often able to identify one or two of these components, thus, achieving a partially successful outbreak investigation. Our data identified investigation characteristics that increase the probability of successful outbreak investigations: a rigorous epidemiology investigation method, a thorough environmental assessment as measured by number of visits to complete the assessment and the collection of clinical samples.

We found that conducting interviews with ill people or a cohort study, compared to not using any epidemiology investigation method, had an increased probability of both completely and at least partially successful outbreak investigations. Both case–control and cohort studies have proven successful in solving foodborne outbreaks, but there are barriers to conducting these during investigations [19, 20]. For example, obtaining information from ill people, as well as finding controls for those ill people, can be challenging, but new investigation guidance from CDC and other strategies developed by state health departments are encouraging [13, 21–23]. For this analysis, the epidemiology investigation method we assigned to each outbreak was the most rigorous investigation method reported by the investigator. In many cases, this most rigorous investigation method was not the sole investigation method used; other methods were used as well. This combination approach may lead to more robust data. Future research efforts should examine the relationship between the number and combination of epidemiology investigation methods and investigation success.

Strategies for collecting information from cases include reviewing credit card receipts, delivery names and addresses to identify who purchased or consumed suspected food items. Strategies like the student-interview model not only relieve some of the burden on health department employees but also are budget-friendly and provide surge support in outbreak settings to ensure complete and accurate data collection from cases [21]. For example, the Minnesota Department of Health trains students from the University of Minnesota, called Team Diarrhoea, on how to conduct phone interviews of reportable enteric pathogen cases identified through surveillance with a detailed questionnaire about illness and exposures [22]. In addition to a student-interview model, the Council to Improve Foodborne Outbreak Response (CIFOR) is a helpful resource on investigating foodborne illness outbreaks. CIFOR also encourages investigators to conduct a case–control or cohort study when possible [24].

Two or more visits to complete the environmental assessment had an increased probability of a completely and at least partially successful outbreak investigation. It is likely that conducting two or more visits to complete assessments provides investigators with a more thorough and comprehensive view of the outbreak and the establishment. For example, a follow-up visit could include interviewing people who were not present at the first visit, observing practices that did not occur during the first visit or focusing observations on key data points based upon new information. This finding is supported by another outbreak data analysis, which found that a quickly initiated assessment and multiple visits to complete the assessment helped identify outbreak contributing factors [11]. Our data suggest that conducting a comprehensive assessment is key to obtaining the information needed for a successful outbreak investigation. Health departments can take advantage of trainings such as the Environmental Assessment Trainings Series (EATS) through CDC to help prepare their investigators to conduct assessments [25].

Collecting clinical samples resulted in an increased probability of a completely and at least partially successful outbreak investigation. Data obtained from clinical samples can identify the pathogen that made people ill; these data can also guide identification of the food item and contributing factors. For example, learning that a pathogen commonly associated with chicken was identified in a clinical stool sample from an ill person may lead the investigator to focus activities on food items made with chicken. A focus group of healthcare professionals and the public identified knowledge, access and cost of clinical samples as issues related to the lack of collecting stool samples in foodborne illness outbreaks [26]. Increased efforts to educate healthcare providers and the public on the importance of the information gained from stool samples may lead to an increase in stool sample collection. Another barrier to collecting stool samples is health departments’ lack of access to laboratory resources, which has been attributed to underfunding [27].

We did not find that environmental sample collection significantly predicted a successful outbreak investigation. However, other evidence suggests that environmental sampling may be a helpful investigation tool. A recent publication highlighted the importance of environmental sampling in retail food establishments, especially if there are barriers to collecting epidemiological and clinical laboratory data. The study, which focused on environmental sampling for norovirus, identified areas retail food workers can target for effective norovirus control and sanitisation [28]. There are also financial barriers to environmental sampling, however, future research could focus on common areas in a retail food establishment where pathogens proliferate and survive. More research and evaluation are needed to fully understand the value of environmental sampling in outbreak investigations.

It is possible that establishment and investigation characteristics related to outbreak investigation success vary by pathogen, as incubation periods, food types and other factors vary by pathogen. Future research is needed to determine if this is the case, and if so, identify the characteristics associated with outbreak investigation success for specific pathogens.

Our study is subject to several limitations. First, NEARS is a voluntary reporting system, and not all health departments in the United States report foodborne outbreaks to NEARS. As such, only a subsample of all outbreaks reported to NORS were included in this study because some did not have a NEARS counterpart. Additionally, it is unknown how many outbreaks are not investigated and reported to CDC. Thus, our findings may not be generalisable to all foodborne illness outbreaks across the country. We also are unable to establish causality, nor can we rule out unassessed variables that may have actually caused the significant associations between variables of interest. There may be some inherent characteristics of outbreaks that influence investigators’ decision to implement a certain investigation characteristic. Another limitation to our data is the introduction of new laboratory methods, such as whole genome sequencing. New laboratory methods may be more readily available to health departments and provide results in a timelier fashion.

This research highlights the importance of epidemiology, environmental health, and laboratory personnel working together to solve foodborne outbreaks. Encouraging open communication and maintaining relationships between epidemiology, environmental health and laboratory partners may increase investigation success. A strong relationship between these partners has been shown to result in a higher rate of outbreak reporting [29]. Nationwide foodborne outbreak investigation efforts continue to develop and provide science-based guidance, such as CIFOR and EATS. This analysis, in addition to past research, highlights the importance of comprehensive outbreak investigations [28, 29].

## Data Availability

Data are available upon request by contacting nears@cdc.gov.

## Supplementary material

For supplementary material accompanying this paper visit http://doi.org/10.1017/S0950268823000237. click here to view supplementary material
