# Supplementary material for: Characteristics associated with successful foodborne outbreak investigations involving United States retail food establishments (2014–2016)
Source: Epidemiol Infect. 2023 Mar 20;151:e78. doi: 10.1017/S0950268823000237 (PMC10204134; doi:10.1017/S0950268823000237)
Supplement: Supplementary file 1 [file S0950268823000237sup001.docx]

**Supplementary Tables**

**Supplementary Table 1. Data collection site by investigation success level— National Environmental Assessment Reporting System, 2014–2016 (N=306)**

|  | **Outbreaks with** | | |
| --- | --- | --- | --- |
| **Site** | **Completely successful investigations*^a^* (n=106, 34.7%)** | **Partially successful investigations*^a^***  **(n=177, 57.8%)** | **Unsuccessful investigations*^a^* (n=23, 7.5%)** |
| A (n=70, 22.9%) | 22 (31.4) | 47 (67.1) | 1 (1.4) |
| B (n=49, 16.0%) | 7 (14.3) | 34 (69.4) | 8 (16.3) |
| C (n=46, 15.0%) | 33 (71.7) | 13 (28.3) | 0 (0.0) |
| D (n=39, 12.8%) | 13 (33.3) | 22 (56.4) | 4 (10.3) |
| E (n=33, 10.8%) | 16 (48.5) | 16 (48.5) | 1 (3.0) |
| F (n=32, 10.5%) | 7 (21.9) | 21 (65.6) | 4 (12.5) |
| G (n=20, 6.5%) | 5 (25.0) | 13 (65.0) | 2 (10.0) |
| H (n=17, 5.6%) | 3 (17.7) | 11 (64.7) | 3 (17.7) |

*^a^*A completely successful outbreak investigation identified an agent, a food item and a contributing factor. A partially successful outbreak investigation identified 1 or 2 of either an agent, a food item, or a contributing factor. An unsuccessful outbreak investigation did not identify an agent, a food item, or a contributing factor.

**Supplementary Table 2. Maximum likelihood estimates for epidemiology investigation method (N=306)**

| Parameter | Level | Estimate | Standard error | 95% CI^d^ | p-value |
| --- | --- | --- | --- | --- | --- |
| Intercept1 | Complete*^a,b^* | -1.74 | 0.59 | (-2.90, -0.57) | 0.003 |
| Intercept2 | At least partially complete*^a,c^* | 1.86 | 0.60 | (0.69, 3.03) | 0.002 |
| Site | B | -1.47 | 0.45 | (-2.36, -0.58) | 0.001 |
|  | C | 1.45 | 0.48 | (0.51, 2.39) | 0.003 |
|  | D | -0.52 | 0.49 | (-1.49, 0.45) | 0.294 |
|  | E | 0.55 | 0.43 | (-0.30, 1.39) | 0.205 |
|  | F | -0.65 | 0.51 | (-1.66, 0.36) | 0.206 |
|  | G | -0.92 | 0.63 | (-2.15, 0.31) | 0.143 |
|  | H | -0.95 | 0.64 | (-2.20, 0.31) | 0.140 |
|  | A | Reference | -- | -- | -- |
| Investigation method | Case control | 1.07 | 0.56 | (-0.03, 2.16) | 0.056 |
|  | Cohort | 1.55 | 0.61 | (0.35, 2.74) | 0.11 |
|  | Interviews | 1.38 | 0.53 | (0.34, 2.42) | 0.009 |
|  | None | Reference | -- | -- | -- |

*^a^*A completely successful outbreak investigation identified an agent, a food item and a contributing factor. A partially successful outbreak investigation identified 1 or 2 of either an agent, a food item, or a contributing factor. An unsuccessful outbreak investigation did not identify an agent, a food item, or a contributing factor.

*^b^*Complete vs. Partially or unsuccessful investigation.

*^c^*At least partially complete vs. unsuccessful investigation.

*^d^*CI = Confidence interval.

**Supplementary Table 3. Least square means for epidemiology investigation method (N=306)**

| Ordinal comparison*^a^* | Epidemiology investigation method | Estimate | Standard Error | p-value | Mean | SEM*^d^* | 95% CI*^e^* |
| --- | --- | --- | --- | --- | --- | --- | --- |
| Complete*^b^* | Case control | -0.98 | 0.27 | <.001 | 0.27 | 0.05 | (0.18, 0.39) |
| Complete*^b^* | Cohort | -0.50 | 0.33 | 0.125 | 0.38 | 0.08 | (0.24, 0.53) |
| Complete*^b^* | Interviews | -0.67 | 0.22 | 0.003 | 0.34 | 0.05 | (0.25, 0.44) |
| Complete*^b^* | None | -2.05 | 0.50 | <0.001 | 0.11 | 0.05 | (0.05, 0.26) |
| At least partially complete*^c^* | Case control | 2.62 | 0.33 | <0.001 | 0.93 | 0.02 | (0.88, 0.96) |
| At least partially complete*^c^* | Cohort | 3.10 | 0.39 | <0.001 | 0.96 | 0.02 | (0.91, 0.98) |
| At least partially complete*^c^* | Interviews | 2.93 | 0.30 | <0.001 | 0.95 | 0.01 | (0.91, 0.97) |
| At least partially complete*^c^* | None | 1.55 | 0.49 | 0.001 | 0.82 | 0.07 | (0.18, 0.39) |

*^a^*A completely successful outbreak investigation identified an agent, a food item and a contributing factor. A partially successful outbreak investigation identified 1 or 2 of either an agent, a food item, or a contributing factor. An unsuccessful outbreak investigation did not identify an agent, a food item, or a contributing factor.

*^b^*Complete vs. Partially or unsuccessful investigation.

*^c^*At least partially complete vs. unsuccessful investigation.

*^d^*SEM = Standard error of the mean.

*^e^*CI = Confidence interval.

**Supplementary Table 4. Odds ratios for epidemiology investigation method (N=306)**

| Epidemiology investigation method | Odds ratio | 95% CI | adjusted 95% CI^a,b^ |
| --- | --- | --- | --- |
| Cohort | 4.70 | (1.42, 15.52) | (0.98, 22.49) |
| Interview | 3.98 | (1.41, 11.27) | (1.02, 15.57) |
| Case control | 2.90 | (0.97, 8.70) | (0.69, 12.22) |
| None | Ref | -- | -- |
|  |  |  |  |
| Cohort | 1.18 | (0.55, 2.55) | (0.43, 3.23) |
| Case control | 0.73 | (0.37, 1.44) | (0.30, 1.78) |
| Interview | Ref | -- | -- |
|  |  |  |  |
| Case control | 0.62 | (0.26, 1.46) | (0.20, 1.91) |
| Cohort | Ref | -- | -- |

^a^p-value adjusted for multiple comparisons using a Scheffé test.

*^b^*CI = Confidence interval.

**Supplementary Table 5. Maximum likelihood estimates for number of visits to complete the environmental assessment (N=306)**

| Parameter | Level | Estimate | Standard error | 95% CI*^d^* | p-value |
| --- | --- | --- | --- | --- | --- |
| Intercept1 | Complete*^a,b^* | -1.32 | 0.40 | (-2.11, -0.54) | <0.001 |
| Intercept2 | At least partially complete*^a,c^* | 2.28 | 0.43 | (1.44, 3.12) | <0.001 |
| Site | B | -0.79 | 0.45 | (-1.67, 0.09) | 0.078 |
|  | C | 1.59 | 0.42 | (0.78, 2.41) | <0.001 |
|  | D | -0.47 | 0.44 | (-1.32, 0.39) | 0.287 |
|  | E | 0.49 | 0.43 | (-0.35, 1.32) | 0.254 |
|  | F | -0.49 | 0.49 | (-1.45, 0.47) | 0.314 |
|  | G | -0.58 | 0.54 | (-1.63, 0.48) | 0.285 |
|  | H | -1.20 | 0.60 | (-2.37, -0.03) | 0.044 |
|  | A | Reference |  |  |  |
| Number of visits | 2+ investigation | 1.25 | 0.44 | (0.38, 2.11) | 0.005 |
|  | One investigation | 0.72 | 0.35 | (0.03, 1.41) | 0.041 |
|  | No investigation | Reference |  |  |  |

*^a^* A completely successful outbreak investigation identified an agent, a food item and a contributing factor. A partially successful outbreak investigation identified 1 or 2 of either an agent, a food item, or a contributing factor. A An un successful outbreak investigation did not identify an agent, a food item, or a contributing factor.

*^b^*Complete vs. Partially or unsuccessful investigation.

*^c^*At least partially complete vs. unsuccessful investigation.

*^d^*CI = Confidence interval.

**Supplementary Table 6. Least square means for number of visits to complete the environmental assessment (N=306)**

| Ordinal comparison*^a^* | Number of visits | Estimate | Standard Error | p-value | Mean | SEM*^d^* | 95% CI*^e^* |
| --- | --- | --- | --- | --- | --- | --- | --- |
| Complete*^b^* | 2+ investigation | -0.25 | 0.31 | 0.416 | 0.44 | 0.08 | (0.23, 0.59) |
| Complete*^b^* | One investigation | -0.79 | 0.19 | <0.001 | 0.31 | 0.04 | (0.24, 0.40) |
| Complete*^b^* | No investigation | -1.50 | 0.31 | <0.001 | 0.18 | 0.05 | (0.11, 0.29) |
| At least partially complete*^c^* | 2+ investigation | 3.34 | 0.39 | <0.001 | 0.97 | 0.01 | (0.93, 0.98) |
| At least partially complete*^c^* | One investigation | 2.81 | 0.27 | <0.001 | 0.94 | 0.01 | (0.91, 0.97) |
| At least partially complete*^c^* | No investigation | 2.09 | 0.32 | <0.001 | 0.89 | 0.03 | (0.82, 0.94) |

*^a^*A completely successful outbreak investigation identified an agent, a food item and a contributing factor. A partially successful outbreak investigation identified 1 or 2 of either an agent, a food item, or a contributing factor. An unsuccessful outbreak investigation did not identify an agent, a food item, or a contributing factor.

*^b^*Complete vs. Partially or unsuccessful investigation.

*^c^*At least partially complete vs. unsuccessful investigation.

*^d^*SEM = Standard error of the mean.

*^e^*CI = Confidence interval.

**Supplementary Table 7. Odds ratios for number of visits to complete the environmental assessment (N=306)**

| Number of visits | Odds ratio | 95% CI | adjusted 95% CI^a,b^ |
| --- | --- | --- | --- |
| 2+ investigation | 3.49 | (1.47, 8.29) | (1.24, 9.82) |
| One investigation | 2.05 | (1.03, 4.08) | (0.90, 4.67) |
| No investigation | Ref | -- | -- |
|  |  |  |  |
| 2+ investigation | 1.70 | (0.84, 3.43) | (0.73, 3.96) |
| One investigation | Ref | -- | -- |

^a^p-value adjusted for multiple comparisons using a Scheffé test.

*^b^*CI = Confidence interval.

**Supplementary Table 8. Maximum likelihood estimates for sample type (N=306)**

| Parameter | Level | Estimate | Standard error | 95% CI*^d^* | p-value |
| --- | --- | --- | --- | --- | --- |
| Intercept1 | Complete*^a,b^* | -1.06 | 0.28 | (-1.60, -0.52) | <0.001 |
| Intercept2 | At least partially complete*^a,c^* | 2.58 | 0.34 | (1.92, 3.24) | <0.001 |
| Site | B | -1.22 | 0.40 | (-2.01, -0.43) | 0.002 |
|  | C | 1.69 | 0.42 | (0.87, 2.51) | <0.001 |
|  | D | -0.36 | 0.44 | (-1.23, 0.50) | 0.411 |
|  | E | 0.38 | 0.43 | (-0.46, 1.22) | 0.377 |
|  | F | -0.74 | 0.46 | (-1.63, 0.16) | 0.107 |
|  | G | -0.73 | 0.54 | (-1.78, 0.32) | 0.176 |
|  | H | -0.99 | 0.61 | (-2.19, 0.21) | 0.106 |
|  | A | Ref | -- | -- | -- |
| Sample type | Both | 1.07 | 0.44 | (0.22, 1.92) | 0.014 |
|  | Clinical | 0.88 | 0.28 | (0.33, 1.43) | 0.002 |
|  | Environmental | 0.53 | 0.57 | (-0.59, 1.65) | 0.357 |
|  | None | Ref | -- | -- | -- |

*^a^*A completely successful outbreak investigation identified an agent, a food item and a contributing factor. A partially successful outbreak investigation identified 1 or 2 of either an agent, a food item, or a contributing factor. An unsuccessful outbreak investigation did not identify an agent, a food item, or a contributing factor.

*^b^*Complete vs. Partially or unsuccessful investigation.

*^c^*At least partially complete vs. unsuccessful investigation.

*^d^*CI = Confidence interval.

**Supplementary Table 9. Least square means for sample type (N=306)**

| Ordinal comparison*^a^* | Sample type | Estimate | Standard Error | p-value | Mean | SEM*^d^* | 95% CI*^e^* |
| --- | --- | --- | --- | --- | --- | --- | --- |
| Complete*^b^* | Both | -0.24 | 0.39 | 0.530 | 0.44 | 0.10 | (0.27, 0.63) |
| Complete*^b^* | Clinical | -0.43 | 0.23 | 0.061 | 0.39 | 0.05 | (0.29, 0.50) |
| Complete*^b^* | Environmental | -0.78 | 0.54 | 0.143 | 0.31 | 0.12 | (0.14, 0.57) |
| Complete*^b^* | None | -1.31 | 0.21 | <0.001 | 0.21 | 0.03 | (0.15, 0.29) |
| At least partially complete*^c^* | Both | 3.34 | 0.45 | <0.001 | 0.97 | 0.01 | (0.93, 0.99) |
| At least partially complete*^c^* | Clinical | 3.21 | 0.32 | <0.001 | 0.96 | 0.01 | (0.93, 0.98) |
| At least partially complete*^c^* | Environmental | 2.86 | 0.57 | <0.001 | 0.95 | 0.03 | (0.85, 0.98) |
| At least partially complete*^c^* | None | 2.33 | 0.25 | <0.001 | 0.91 | 0.02 | (0.86, 0.94) |

*^a^*A completely successful outbreak investigation identified an agent, a food item and a contributing factor. A partially successful outbreak investigation identified 1 or 2 of either an agent, a food item, or a contributing factor. An unsuccessful outbreak investigation did not identify an agent, a food item, or a contributing factor.

*^b^*Complete vs. Partially or unsuccessful investigation.

*^c^*At least partially complete vs. unsuccessful investigation.

*^d^*SEM = Standard error of the mean.

*^e^*CI = Confidence interval.

**Supplementary Table 10. Odds ratios for sampling type (N=306)**

| Sample type | Odds ratio | 95% CI | adjusted 95% CI^a,b^ |
| --- | --- | --- | --- |
| Both | 2.91 | (1.24, 6.83) | (0.95, 8.90) |
| Clinical | 2.41 | (1.39, 4.20) | (1.17, 4.99) |
| Environmental | 1.69 | (0.55, 5.19) | (0.39, 7.35) |
| None | Ref | -- | -- |
|  |  |  |  |
| Both | 1.72 | (0.49, 6.08) | (0.33, 9.01) |
| Clinical | 1.42 | (0.45, 4.47) | (0.32, 6.38) |
| Environmental | Ref | -- | -- |
|  |  |  |  |
| Both | 1.21 | (0.50, 2.89) | (0.38, 3.80) |
| Clinical | Ref | -- | -- |

^a^p-value adjusted for multiple comparisons using a Scheffé test.

*^b^*CI = Confidence interval.
